# High imatinib dose overcomes insufficient response associated with ABCG2 haplotype in chronic myelogenous leukemia patients

**DOI:** 10.18632/oncotarget.1050

**Published:** 2013-07-18

**Authors:** Marc Delord, Philippe Rousselot, Jean Michel Cayuela, François Sigaux, Joëlle Guilhot, Claude Preudhomme, François Guilhot, Pascale Loiseau, Emmanuel Raffoux, Daniela Geromin, Emmanuelle Génin, Fabien Calvo, Heriberto Bruzzoni-Giovanelli

**Affiliations:** ^1^ Plateforme de Bioinformatique et Biostatistique, Institut Universitaire d'Hématologie, Université Paris Diderot, Sorbonne Paris Cité; ^2^ Service d'Hématologie et d'Oncologie, H&ocirc;pital Mignot, Université Versailles Saint-Quentin-en-Yvelines; ^3^ Laboratoire Central d'Hématologie, Hôpital Saint Louis; ^4^ EA3518, Université Paris Diderot, Sorbonne Paris Cité; ^5^ Inserm CIC 0802, CHU de Poitiers, Poitiers; ^6^ Laboratoire d'Hématologie, Inserm, U837, CHRU et Université de Lille Nord, Institut de Recherche sur le Cancer de Lille; ^7^ Service d'Immunologie et Histocompatibilité et INSERM U940, Hôpital Saint Louis; ^8^ Service des Maladies du Sang, Hôpital Saint Louis; ^9^ Plateforme de Ressources Biologiques, Hôpital Saint Louis, Paris; ^10^ Inserm U1078, CHU Brest, Université Bretagne Occidentale, Brest; ^11^ Pharmacologie, Université Paris Diderot, Sorbonne Paris Cité, Paris; ^12^ Centre d'Investigations Cliniques 9504 INSERM-AP-HP, Hôpital Saint-Louis, Paris, France

**Keywords:** CML, Imatinib, SNPs, ABCG2, Pharmacogenetic, molecular response, BCR-ABL

## Abstract

Pharmacogenetic studies in chronic myelogenous leukemia (CML) typically use a candidate gene approach. In an alternative strategy, we analyzed the impact of single nucleotide polymorphisms (SNPs) in drug transporter genes on the molecular response to imatinib, using a DNA chip containing 857 SNPs covering 94 drug transporter genes. Two cohorts of CML patients treated with imatinib were evaluated: an exploratory cohort including 105 patients treated at 400 mg/d and a validation cohort including patients sampled from the 400 mg/d and 600 mg/d arms of the prospective SPIRIT trial (n=239). Twelve SNPs discriminating patients according to cumulative incidence of major molecular response (CI-MMR) were identified within the exploratory cohort. Three of them, all located within the ABCG2 gene, were validated in patients included in the 400 mg/d arm of the SPIRIT trial. We identified an ABCG2 haplotype (define as G-G, rs12505410 and rs2725252) as associated with significantly higher CI-MMR in patients treated at 400 mg/d. Interestingly, we found that patients carrying this ABCG2 “favorable” haplotype in the 400 mg arm reached similar CI-MMR rates that patients randomized in the imatinib 600 mg/d arm. Our results suggest that response to imatinib may be influenced by constitutive haplotypes in drug transporter genes. Lower response rates associated with “non-favorable” ABCG2 haplotypes may be overcome by increasing the imatinib daily dose up to 600 mg/d.

## INTRODUCTION

Imatinib (Glivec®, Novartis Pharmaceuticals Corporation), along with other tyrosine kinase inhibitors (TKIs), has revolutionized treatment of chronic myelogenous leukemia (CML). Long-term follow-up of the IRIS pivotal study revealed that overall survival for patients who received imatinib as initial therapy was as high as 88% at 6 years [1]. Two second generation TKIs, dasatinib (Sprycel®, Bristol-Myers Squib) and nilotinib (Tasigna®, Novartis Pharmaceuticals Corporation) are now registered as frontline therapy for chronic phase CML (CP-CML) patients. Recent studies reported faster and deeper responses assessed by cytogenetic or molecular analysis with these drugs [2-3], however data are yet too preliminary to determine whether these agents will offer a survival advantage over imatinib. Comorbidities, age, and co-medications tend to drive the choice of the TKI in first-line therapy. Emerging reports of adverse events with nilotinib (peripheral arterial occlusive disease) [4] and dasatinib (pulmonary hypertension) [5] along with the upcoming arrival of generic imatinib will move TKI therapy towards a personalized approach. Disease-related factors such as initial Sokal score have been shown to influence molecular responses [6-9], however, few patient-related parameters such as adherence to therapy or trough imatinib levels have been evaluated [10-12].

Association studies have suggested that single nucleotide polymorphisms (SNPs) may be related to a susceptibility to develop CML [13-16], CML progression [17-18] or intracellular accumulation of imatinib in leukocytes of CML patients [19]. Only a limited number of selected SNPs have been tested in relation to imatinib response[20-28], and results obtained with the most extensively studied gene ABCG1 (MDR1) are not consistent across published analyses[29-30]. Complete cytogenetic response (CCR) at 12 months remains the best surrogate marker for survival in CML patients. However, major molecular response (MMR, defined by BCR-ABL≤0.1%) was used as a primary end point of clinical trial (such as in the ENEST 1^st^ study) [31] and MMR at 18 months is also part of the “optimal response” definition of the ELN2009 recommendation for CML patient management [32]. We thus selected cumulative incidence of major molecular response (CI-MMR) as a criteria to identify SNPs in drug transporter genes which are associated with a favorable outcome. We performed an association study using a custom-made DNA chip in an exploratory cohort. An ABCG2 haplotype associated with high CI-MMR was identified. We then validated this haplotype in an independent “prospective-retrospective” cohort[33], and evaluated its impact according to imatinib daily dose.

## RESULTS

Patient characteristics are presented in Table 1. Median age, sex ratio and Sokal score distribution were comparable between all cohorts (*P* > 0.05 in all cases).

**Table 1:** Patient characteristics

		Saint-Louis Exploratory Cohort (SLEC)	SPIRIT Validation Cohort (SVC)			Total
		(400 mg) N=105	(400 mg) N=132	(600 mg) N=107	(SVC) N=239	N=344
**Gender**	Male	63 (60%)	88 (67%)	52 (49%)	143 (60%)	206 (60%)
	Female	42 (40%)	44 (33%)	55 (51%)	96 (40%)	138 (40%)
**Sokal Score**	Low	34 (32%)	52 (39%)	38 (36%)	90 (38%)	124 (36%)
	Int.	24 (23%)	54 (41%)	45 (42%)	99 (41%)	123 (36%)
	High	15 (14%)	26 (20%)	24 (22%)	50 (21%)	65 (19%)
	NA*	32	0	0	0	32
**Median age (years)**		50.5	51.8	51.5	51.5	51.5

All p values of differences among groups were not significant

* Not available

### Determinants of CI-MMR

**Figure 1:**
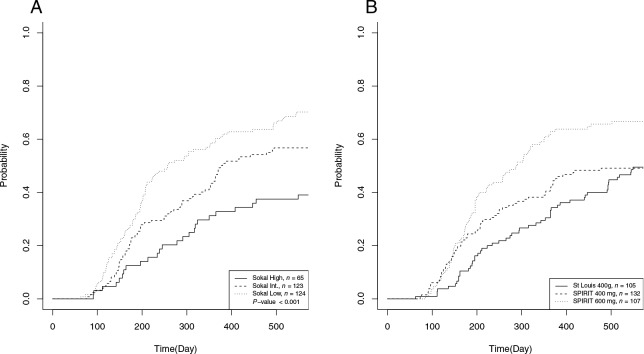
Cumulative incidence of MMR (CI-MMR) according to Sokal score and treatment arms A) 18 months CI-MMR was estimated with respect to Sokal score (*n =* 312). A Fine and Gray model showed that time to MMR was related to Sokal status and that the coefficient of regression within the first 18 months decreased by 36% (95% confidence interval (CI), 47% to 22%) on average when Sokal increased (*P* < 0.001). CI-MMR was 70% for the low Sokal score, 57% for the intermediate Sokal score and 39% for high Sokal score. B) CI-MMR was estimated in the exploratory cohort (SLEC) and compared to both treatment arms of the validation cohort (SVC). CI-MMR was comparable between the SLEC and the 400 mg/d arm of SVC (n = 237, *P* = 0.700), but significantly different between the SLEC and the 600 mg/d arm of SVC (n = 212, *P* = 0.003). HR was 1.71% (95% CI, 1.20% to 2.44%) in the latter (*n =* 212, *P =* 0.003). CI-MMR was 49% for the exploratory cohort, 49% and 67% for the 400 and the 600 mg/d arm of the SVC, respectively.

CI-MMR were estimated according to Sokal score (*n =* 312). Using the Fine and Gray model we confirmed that CI-MMR was strongly related to Sokal score levels (regression coefficient: 0.64, 95% confidence interval (CI), 0.53 to 0.78, *P* < 0.001). Figure 1A illustrates the inverse relationship between Sokal score and CI-MMR.

CI-MMR was estimated in the SLEC (*n* = 105), SVC (*n* = 239), and in the two imatinib SVC treatment arms (*n* = 132 at 400 mg/d; *n* =107 at 600 mg/d; Figure 1B). CI-MMR was comparable between the SLEC and the SVC 400 mg/d arm (*P =* 0.700), but was significantly higher in the SVC 600 mg/d arm (*P* = 0.003). The regression coefficient increased by more than 50% (1.53, 95% CI, 1.09 to 2.14) when the dose increased from 400 mg/d to 600 mg/d (*P* = 0.014).

### SNP association study

Of the 857 selected SNPs, 413 (48.2%) from 86 drug transporters were well genotyped in all evaluated patients and passed quality control criteria. We identified 12 SNPs (located in eight transporter genes) in the SLEC group which were significantly associated with CI-MMR at 18 months on the basis of an FDR < 50% (Table 2). Only one of these SNPs (rs12505410), located in the ABCG2 gene, was significantly associated with CI-MMR in the overall SVC cohort. Separate analysis of the two SVC cohorts revealed three SNPs (rs12505410, rs13120400 and rs2725252), all from the ABCG2 gene, which were significantly associated with response in the 400 mg/d arm while none were associated in the 600 mg/d arm. CI-MMR at 18 months in the two SVC groups for the three validated ABCG2 SNPs is shown in Supplementary Figure SF1.

**Table 2:** Drug transporter SNPs associated with CI-MMR at 18 months

SNP	Chr.	Coordinate	Transporter gene symbol	SLEC	SVC (P-value)			
				P-value	FDR	All	400 mg/d	600 mg/d
rs609468	6	160498904	SLC22A1	<0.001	0.001	0.920	0.210	0.090
rs10841907	12	21942563	ABCC9	0.001	0.238	0.310	0.670	0.450
rs12505410	4	89249865	**ABCG2**	0.002	0.238	0.045*	0.035*	0.320
rs4149182	11	62524689	SLC22A8	0.002	0.238	0.750	0.640	0.820
rs1189451	13	94520087	ABCC4	0.005	0.430	0.260	0.360	0.330
rs17556915	14	69318111	SLC10A1	0.008	0.482	0.068	0.130	0.230
rs11024300	11	17452549	ABCC8	0.009	0.482	0.870	0.550	0.160
rs13120400	4	89252551	**ABCG2**	0.012	0.482	0.140	0.046*	0.740
rs2725252	4	89280934	**ABCG2**	0.012	0.482	0.086	0.047*	0.740
rs2665691	11	22327832	SLC17A6	0.012	0.482	0.075	0.110	0.360
rs1678405	13	94627682	ABCC4	0.014	0.482	0.710	0.850	0.370
rs1048099	11	17453092	ABCC8	0.014	0.482	0.950	0.220	0.150

Chr., chromosome; FDR, false discover rate; SNP, single nucleotide polymorphism

* Significant association between CI-MMR and SNP (P < 0.05)

### Haplotype frequency

As expected, pairwise linkage disequilibrium was found between the three SNPs at the ABCG2 locus in the SLEC. We therefore performed haplotyping at this locus. Estimated frequencies was 22% for haplotype 1 (G-C-G) having G, C and G bases at loci rs12505410, rs13120400 and rs2725252, respectively, < 1% for haplotype 2 (G-C-T), 5% for haplotype 3 (G-T-G), 15% for haplotype 4 (T-T-G), 57% for haplotype 5 (T-T-T) and 2% for haplotype 6 (G-T-T) (Supplementary Table ST2).

**Figure 2:**
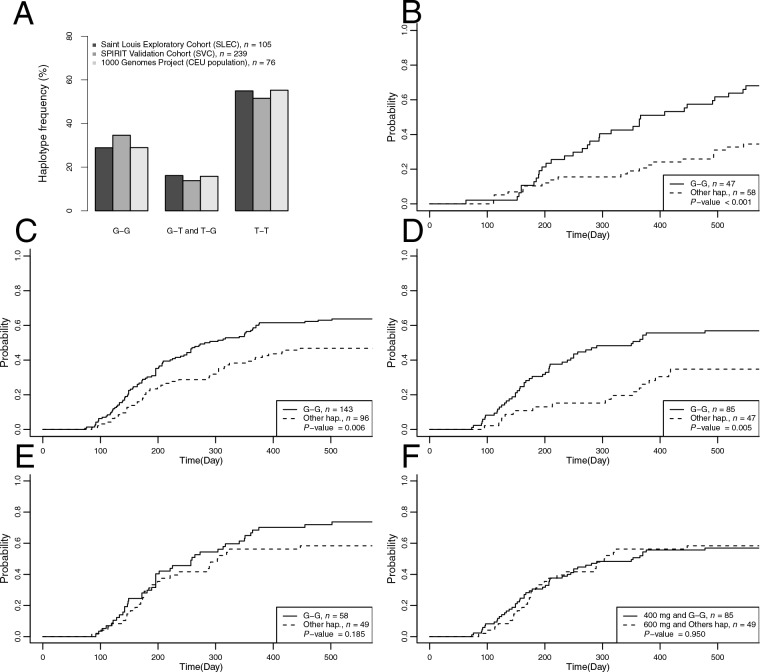
Frequencies and cumulative incidence of MMR relative to ABCG2 haplotypes A) Distribution of haplotype frequencies in the SLEC, SVC and the CEU populations. Haplotypes were distributed homogeneously over the different populations. B) Cumulative incidence at 18-months of major molecular response (CI-MMR) was calculated in the SLEC according to ABCG2 haplotypes G-G. CI-MMR of patients with at least one copy of haplotype G-G was 69%. CI-MMR for other patients was 34%. C) CI-MMR at 18-months in all SVC patients with haplotype G-G was 63% and 47% for other patients (P = 0.006). D) CI-MMR in SVC patients treated with 400 mg/d was 57% and 36% for G-G haplotype carriers and other haplotype carriers respectively (P = 0.005). E) CI-MMR in SVC patients treated with 600 mg/d was 74% and 58% for G-G haplotype carriers and other patients respectively (P = 0.185). F) CI-MMR was not significantly different between in SVC patients with haplotype G-G receiving 400 mg/d and those with other haplotypes receiving 600 mg/d (57% vs 58% respectively, P = 0.950).

Haplotype frequencies at the same loci were also calculated in the independent set of unrelated individuals from the CEU population (*n* = 76). Frequency of haplotype 1 plus 3 was 27% in the SLEC *versus* 28% in the CEU population, 56% *versus* 55% for haplotype 5 and 16% *versus* 17% for other haplotypes. Equal distribution of haplotypes between populations was confirmed (*P* = 0.70; Figure 2A and Supplementary Table ST2).

### Haplotype association study

CI-MMR at 18 months was evaluated with respect to ABCG2 haplotype distribution in the exploratory cohort. Multivariate analysis identified haplotypes 1 and 3 (G-C-G and G-T-G respectively) as linked to significantly higher CI-MMR rates. These two haplotypes share the same alleles at rs12505410 and rs2725252 and differ at rs13120400, suggesting that the association could be with the G-G haplotype at these two SNPs; carriers of the G-G haplotype at rs12505410 and rs2725252 had significantly higher CI-MMR than non-carriers (Figure 2B).

The validation cohort was analyzed using the same approach. Haplotype frequencies at ABCG2 locus were verified as being comparable to the SLEC and the CEU populations (Figure 2A). This confirmed the analysis in the SVC population was performed according to the same assumptions as for the exploratory cohort and was analyzed overall and by treatment arms (Figure 2 C, D, E and F and Table 3).

**Table 3:** ABCG2 haplotype associated with CI-MMR at 18 months

Univariate		SLEC		SVC					
				All patients		400 mg/d		600 mg/d	
		n = 105	(P-value)	n = 239	(P-value)	n = 132	(P-value)	n = 107	(P-value)
G-G		68.09	(<.001)	63.72	(.006)	56.90	(.005)	73.68	(.185)
Other haplotypes		34.48		46.81		34.78		58.33	
**Bivariate Fine and Gray model statistics**		**n = 73**		**n = 239**		**n = 132**		**n = 107**	
G-G	Reg. Coef.	2.27	(.006)	1.75	(.002)	2.41	(.002)	1.32	(.270)
	95%CI	1.26 to 4.10		1.22 to 2.51		1.39 to 4.19		0.81 to 2.15	
Sokal score	Reg. Coef.	0.64	(.024)	0.62	(<.001)	0.68	(.034)	0.51	(.001)
	95%CI	0.43 to 0.94		0.49 to 0.78		0.47 to 0.97		0.38 to 0.69	

In the overall SVC, 18-month CI-MMR increased by 36% for patients with one copy of haplotype G-G (*P* = 0.005, Figure 2C). In patients included in the imatinib 400 mg/d arm, 18-month CI-MMR increased by 64% (*P* = 0.002, Figure 2D). Association of haplotype G-G with CI-MMR in patients included in the imatinib 600 mg/d arm was not significant (*P* = 0.180, Figure 2E). CI-MMR curves of patients with haplotype G-G included in the imatinib 400 mg/d arm were comparable to those of patients with other haplotypes in the imatinib 600 mg/d arm (Figure 2F, *P* = 0.480). Of note, Sokal score remained an independent determinant of CI-MMR in all populations in a multivariate model (Table 3).

As expected from results of the Fine and Gray model in the validation cohort, early molecular responses (BCR-ABL^IS^ at 3 months ≤ 10%) as well as responses of interest (BCR-ABL^IS^ at 12 months ≤ 1%, BCR-ABL^IS^ at 18 months ≤ 0.1%) were associated with the ABCG2 G-G haplotype in patients treated with imatinib 400 mg/d (Table 4).

**Table 4:** Association between ABCG2 haplotype and molecular response in the SVC

		BCR-ABLIS ≤ 10% at 3 months			BCR-ABLIS ≤ 1% at 12 months			BCR-ABLIS ≤ 0.1% at 18 months		
Imatinib dose	Response	Haplotype G-G	Others haplotypes	P-value	Haplotype G-G	Others haplotypes	P-value	Haplotype G-G	Others haplotypes	P-value
400mg	Yes	52	14	0.001*	53	19	0.025*	48	16	0.022*
	No	33	33		32	28		37	31	
600mg	Yes	41	28	0.209	43	31	0.316	41	28	0.209
	No	17	21		15	18		17	21	

* Significant association between ABCG2 haplotype and molecular response (P < 0.05)

Using the CEU population genotypes, we tested associations between haplotype G-G and 1772 SNPs near or within the ABCG2 gene (including rs2231135, rs2231137 and rs2231142). We identified 240 SNPs with alleles in linkage disequilibrium with the haplotype G-G. Interestingly rs2231135, located in the 5'UTR and potentially linked to ABCG2 differential expression, was significantly linked to haplotype G-G (*P* = 0.043).

### DISCUSSION

We report here a haplotype/CI-MMR association study in two independent cohorts of CP-CML patients receiving imatinib, in which patients were genotyped using a custom-made DNA chip [34] mainly containing tag SNPs. The exploratory SLEC cohort reflected real-life practice with patients treated at imatinib 400 mg/d, while the validation SVC cohort was composed of patients included in the SPIRIT clinical trial and randomized to imatinib 400 mg/d or imatinib 600 mg/d. We used the first cohort to perform an association study with a large number of drug transporter genes SNPs in CP-CML patients and the second cohort in order to validate these results.

Studies evaluating SNPs in CML patients have been performed on a small number of genes pre-selected for their potential relationship to response [20-28]. However, the clinical significance of these results is far from established, firstly because different genes were analyzed by different groups and secondly when the same gene was studied, the SNPs analyzed were not the same. Accordingly, only two genes have been tested in a validation cohort. One of them was confirmed (IFN-γ) [25] whereas results for ABCG1 were not reproducible or were contradictory [26-30].

Our approach confirmed known key data: the inverse relationship between MMR rates and Sokal score and significantly higher MMR rates at 12 and 18 months with 600 mg imatinib compared to 400 mg [35-36]. We have extended these results with the identification of an ABCG2 haplotype associated with significantly higher CI-MMR in two patient groups receiving 400 mg/d imatinib (i.e. a real-life and a clinical setting). Patients with at least one copy of haplotype G-G (G at rs12505410 and G at rs2725252) were good responders at 400 mg. This “favorable” haplotype is widespread; about half of the population in our study carries at least one copy. Interestingly, patients in the validation cohort treated at 600 mg/d who did not carry this haplotype showed similar CI-MMR levels as haplotype carriers treated at 400 mg/d. Moreover, the clinical pertinence of these results is supported by the association of the G-G ABCG2 haplotype with the early molecular response at 3 months and responses of interest at 12 and 18 months.

The ATP-binding cassette transporter ABCG2 (BCRP, MXR or ABCP) is highly expressed in the gastrointestinal tract and liver, and is involved in absorption, distribution and excretion of a wide variety of clinically relevant drugs, among them imatinib [37-38]. Germline polymorphisms in the ABCG2 gene have been described as affecting expression, cellular localization and/or substrate recognition of the encoded protein. More than 24 sequence variations have been reported. The most studied C421A (rs2231142) nucleotide change, results in a glutamine-to-lysine substitution in the translated protein (pQ141K). Among the studies of SNPs in the ABCG2 gene, two included CML patients treated with imatinib [20, 26]. The first showed that the homozygous GG genotype of rs2231137 in ABCG2 in advanced stage CML patients was significantly associated with poor major or complete cytogenetic response[26], although this result was not subsequently validated in an independent cohort 20.

We were able to test the association between ABCG2 haplotype G-G and 1772 other ABCG2-related SNPs including rs2231137 and rs2231142 (not represented in our DNA chip) by means of an open-access database. We found that rs2231135, which is a 5'UTR and is potentially implicated in ABCG2 expression, was associated with haplotype G-G of ABCG2. However, the statistical association between ABCG2 polymorphisms and CI-MMR does not necessary imply a causal relationship and eventual changes in ABCG2 expression linked to these polymorphisms will have to be addressed in further studies.

The SNP rs609468 from SLC22A1 (also known as OCTN1 or HOCT1 and involved in imatinib uptake) had the lowest p-value in our exploratory cohort (SLEC). SLC22A1 has previously been studied in relation to imatinib response. The SNP rs683369 and advanced disease stage correlated with a high rate of loss of cytogenetic response or treatment failure to imatinib [26], whereas a polymorphism in rs1050152 was significantly associated with MMR [20]. SLC22A1 activity was found predictive of MMR and correlated with overall and event-free survival, especially in patients receiving less than 600 mg/d of imatinib daily [39]. Although the hypothesis that changes in SLC22A1 sequence may result in changes of activity affecting imatinib bioavailability is particularly attractive, more recent studies have failed to demonstrate association between SLC22A1 SNPs and imatinib response [27, 40]. In the same way, SNP rs609468 identified in the SLEC, was not validated in the SVC in our study.

Finally, only 3 SNPs out of 12 were validated. This result is in accordance with the FDR level selected in our analysis. Interestingly no SNPs from our chip located in the ABCG1 gene were found to be associated with imatinib response in our analysis.

Pharmacological studies have suggested that imatinib trough levels may mediate molecular response [10, 12], and it is thus of interest to identify if this is the case for the observed effect of ABCG2 haplotype on molecular response (as is the dose effect). This question will be addressed in patients included in the OPTIM-imatinib clinical trial, an ongoing prospective clinical trial evaluating imatinib dose adjustment driven by imatinib trough levels (OPTIM-Imatinib, EudraCT number 2010-019568-35).

In conclusion, our results demonstrate the influence of a constitutive ABCG2 haplotype on the response to imatinib in CP-CML patients and raise the possibility of personalizing imatinib daily doses in this population on the basis of constitutive genotyping.

## PATIENTS AND METHODS

### Study design

This study was approved by the Human Ethics Committee of the St Louis Hospital, Paris. Written informed consent was obtained from all patients prior to study participation.

Analyses were performed in two independent patient cohorts; the Saint Louis exploratory cohort (SLEC) treated at the Saint Louis hospital specialized clinical trial center (CIC), and the SPIRIT validation cohort (SVC) treated at participating centers in the SPIRIT trial [36] (clinicaltrials.gov: NCT00219739). Data were collected at participating institutions, analyzed using the sponsor's data management systems. Access to primary clinical data was available to all authors.

### Patients and assessments

The SLEC included 105 consecutively referred CP-CML patients enrolled at the CIC between 2006 and 2009 and treated with imatinib 400 mg/d. The SVC included 239 CP-CML patients from the prospective SPIRIT trial based on sample availability from patients recruited to the imatinib arms; 132 patients were treated with 400 mg/d and 107 with 600 mg/d. Patients receiving imatinib plus pegylated interferon-alpha2b or imatinib plus cytarabine were not analyzed.

Peripheral blood samples for DNA extraction were collected at the time of recruitment. BCR-ABL transcripts were quantified by RTQ-PCR every 3 months in accordance with international recommendations, and expressed according to the international scale (IS) as a BCR-ABL/ABL standardized ratio (BCR-ABL^IS^) [41]. MMR was defined as a BCR-ABL^IS^ ratio ≤0.1%).

Genotypes from 76 unrelated and unaffected individuals obtained from the CEU population (Utah residents with northern and western European ancestry) from the CEPH (“Centre d'Etudes du Polymorphisme Humain”) database were downloaded from the 1000 Genomes Project website [42].

### SNP selection and genotyping

A dedicated DNA chip[34] designed in 2006 by the French REPAC network (coordinated by Pierre Laurent-Puig and Fabien Calvo, Supplementary Table ST1) was used for patient genotyping. Among 16 561 SNPs on the chip, 857 covering 94 drug transporter genes were selected. Genotyping was performed by Integragen SA using the Illumina GoldenGate assay. A list of variations from the ABCG2 gene was downloaded from the 1000 Genomes Project website [34]. All 16 561 SNPs genotyped in the 105 SLEC patients and the 239 SVC patients were included in the quality control process. Individuals with a call rate below 90%, SNPs with minor allele frequency below 10%, and SNPs with a call rate below 90% were excluded.

### Statistical analysis

Patient characteristics were compared between cohorts using Chi-squared or Wilcoxon tests. Sample size simulations show that for conventional type 1 and 2 error rates, in a population of about one hundred with approximately 60% of patients expected to reach MMR within the first 18 months and with a regression coefficient of two between groups, SNPs differentiating more than one-third of patients are required [44]. This result led us to investigate those SNPs discriminating at least one third of the patients in a recessive mode.

CI-MMR at 18 months was analyzed in the various patient cohorts (SLEC/SVC, and by imatinib dose) using the Fine and Gray regression model with multivariate and univariate analyses. Adverse events, toxicities or deaths not related to CML which led to loss of molecular follow-up were handled as competing events. An SNP association analysis for CI-MMR at 18 months was also performed using the Fine and Gray model. Markers with a false discovery rate (FDR) < 50% in the SLEC population were investigated in the validation cohort (overall, 400 mg/d and 600 mg/d) using a significance cut-off of *P* = 0.05. The Benjamini and Hochberg method was used for multiple testing issues [45]. Haplotype frequencies were estimated in the SLEC, SVC (overall, 400 mg/d and 600 mg/d) and CEU populations using the classic EM algorithm on unrelated individuals implemented in the Haplo.stats R library [46-48]. Homogeneity in haplotypic distribution between populations was tested. An association analysis between haplotype or Sokal score with CI-MMR at 18-months was performed using a Fine and Gray model. The association between early molecular response (BCR-ABL^IS^ ≤ 10% at 3 months) and responses of interest (BCR-ABL^IS^ ≤ 1% at 12 months and BCR-ABL^IS^ ≤ 0.1% at 18 months) and ABCG2 haplotypes were tested using the chi-squared test. Data were analyzed using R Project for Statistical Computing software (R version 2.15.2) [49].

Supplementary information is available on the Oncotarget website.

## Supplementary File


