# Supplementary material for: High imatinib dose overcomes insufficient response associated with ABCG2 haplotype in chronic myelogenous leukemia patients
Source: Oncotarget. 2013 Jul 18;4(10):1582–91. doi: 10.18632/oncotarget.1050 (PMC3858547; doi:10.18632/oncotarget.1050)
Supplement: Supplementary file 1 [file oncotarget-04-1582-s001.doc]

**SUPPLEMENTARY DATA**

**Supplementary Table ST1. Eligible SNPs from drug transporter genes on the REPAC DNA chip (SNPs, n = 857; Genes, n = 94)**

| **Gene symbol** | **Chromosome** | **SNP name** | **Frequency** |
| --- | --- | --- | --- |
| ABCA1 | 9 | rs363717, rs2274873, rs12686004, rs1800977, rs2422493 | 5 |
| ABCA10 | 17 | rs1014235, rs1011789, rs6501291, rs6501292, rs11871771, rs7222780, rs1034878, rs1558168, rs997639 | 9 |
| ABCA11 | 4 | rs6810900 | 1 |
| ABCA12 | 2 | rs4672734, rs2274412, rs17492577, rs1464678, rs7579031, rs4533467, rs10498030, rs10198064, rs7575071, rs16853200, rs10187636, rs1403826, rs2372479, rs10498032, rs1568742, rs2970958, rs2948974, rs12464205, rs2948983 | 19 |
| ABCA13 | 7 | rs1526106, rs10252745, rs4917139, rs6957787, rs7789339, rs1526104, rs1880740, rs1880738, rs1526095, rs10499674, rs2222648, rs12673367, rs6969141, rs3931814, rs4024044, rs12056015, rs11772384, rs6948541, rs1526093, rs2163938, rs10278745, rs10236551, rs1865195, rs7778020, rs2060330, rs6945363, rs6973815, rs6943378, rs4917133, rs1433515, rs11770110, rs10236459, rs1030708, rs4917157, rs2362310, rs1997238, rs1881078, rs10231745, rs12113960, rs10276788, rs10238373, rs10254535, rs10268266 | 43 |
| ABCA3 | 16 | rs2238464, rs2240523, rs323072, rs11867129, rs323066 | 5 |
| ABCA4 | 1 | rs7537325, rs537831, rs945067, rs12083701, rs2275033, rs3818778, rs914958, rs915200, rs915199, rs17110850, rs17110858, rs10493867, rs4147848, rs472908, rs11165065, rs11165068, rs1889548, rs11165069, rs4147839, rs553608, rs1191232, rs4140392, rs549114, rs12758774, rs2151848, rs3112831, rs4147830, rs17461953, rs4847196, rs554931, rs952499, rs4147825, rs4147823, rs4147822, rs12088309, rs548122, rs950283, rs4147812, rs11165074, rs3789440, rs10782976, rs7535005, rs2184339, rs1931572 | 44 |
| ABCA5 | 17 | rs12449649, rs486840, rs11871086, rs333937, rs571598 | 5 |
| ABCA6 | 17 | rs7504026, rs4968838 | 2 |
| ABCA7 | 19 | rs3764650, rs3752237, rs3752240, rs3752243, rs3752246, rs4147932, rs2242436 | 7 |
| ABCA8 | 17 | rs11652518, rs3744493, rs4246431, rs4147997, rs6501851, rs4147994, rs4147993, rs4147988, rs4147976, rs3744498, rs4147967 | 11 |
| ABCA9 | 17 | rs764426, rs7207125, rs2302291, rs10512523, rs11871635 | 5 |
| ABCB1 | 7 | rs3842, rs1045642, rs4148740, rs10280101, rs7787082, rs2032583, rs11983225, rs10248420, rs2235040, rs12720067, rs2235013, rs2235033, rs1128503, rs868755, rs2235015 | 15 |
| ABCB10 | 1 | rs927204, rs12734075, rs1020353 | 3 |
| ABCB11 | 2 | rs473351, rs483234, rs17540154, rs853774, rs3755160, rs4148797, rs17267869, rs3770585, rs2058996, rs7602171, rs3770602 | 11 |
| ABCB4 | 7 | rs12154399, rs31666, rs31670, rs1558376, rs2302387, rs4148808, rs4148805 | 7 |
| ABCB5 | 7 | rs10216013, rs6950237, rs4721927, rs2285560, rs1011559, rs13240297, rs11764760, rs17817117, rs10230205, rs7805806, rs2190410, rs11974407, rs12700229, rs17143272, rs2024046, rs2158855, rs10230796, rs12673841, rs6461511, rs1023541, rs10255466, rs10950831, rs4721941, rs6959944, rs9638774, rs6461515, rs10231520, rs12669866, rs6461517, rs13225659 | 30 |
| ABCB6 | 2 | rs1109866 | 1 |
| ABCB8 | 7 | rs6944935, rs6947821, rs2303924, rs4148850, rs2303928 | 5 |
| ABCC1 | 16 | rs504348, rs215101, rs4148333, rs215098, rs215052, rs12922744, rs246217, rs12927980, rs4780589, rs16967145, rs246214, rs152033, rs246240, rs875740, rs903880, rs8054670, rs246227, rs173476, rs35587, rs35592, rs35593, rs17287570, rs35600, rs35601, rs35605, rs4148351, rs152029, rs4148354, rs2889517, rs3888565, rs11861085, rs11864374, rs3887893, rs3743527, rs212090, rs212091, rs212093, rs212094, rs12448760 | 39 |
| ABCC10 | 6 | rs831314, rs9349256, rs2185631, rs2487663, rs2125739 | 5 |
| ABCC11 | 16 | rs10163354, rs12443685, rs11866529, rs17822931 | 4 |
| ABCC12 | 16 | rs7193955, rs12149826, rs8046826 | 3 |
| ABCC13 | 21 | rs2822519, rs2822520, rs8129984, rs17273981, rs2822522, rs4816274, rs2822531, rs2254297, rs2822545, rs2822548, rs1153303, rs2822553, rs1014539, rs2822556, rs1153312, rs9974847, rs1153322, rs1153333, rs2822567, rs1297152, rs1123271 | 21 |
| ABCC2 | 10 | rs717620, rs2756103, rs2756109, rs2273697, rs11190291, rs2002042, rs4148398, rs3740066, rs3740065 | 9 |
| ABCC3 | 17 | rs2189595, rs8073706, rs1989983, rs2412333, rs739921, rs739922, rs1541392, rs12051822, rs4794173, rs4148411, rs4148412, rs739923, rs16949202, rs8075406, rs2277624, rs3785911, rs1051640, rs17563146 | 18 |
| ABCC4 | 13 | rs34559063, rs4148553, rs4148551, rs3742106, rs6492763, rs2182262, rs1151471, rs10219913, rs12854072, rs3782945, rs1189457, rs12853814, rs1189451, rs12853981, rs1751051, rs1617785, rs12584534, rs2766481, rs9561784, rs1729741, rs1751070, rs10508023, rs1189470, rs1479390, rs7988595, rs1678396, rs1750996, rs9561797, rs12429339, rs17189376, rs1678405, rs1887162, rs1564352, rs4773843, rs1611822, rs1678374, rs9524821, rs2487566, rs2274403, rs9516530, rs1751022, rs12429872, rs2274410, rs9634642, rs4148454, rs9516546, rs2892716, rs4148434, rs12584649, rs9516551, rs4771910, rs4773867, rs10508017, rs4148422, rs7324283, rs7330673, rs4771912, rs4773875, rs17189561, rs8001444 | 60 |
| ABCC5 | 3 | rs1554395, rs1016752, rs3817404, rs939336, rs7636910, rs10937157, rs10937158, rs16858328 | 8 |
| ABCC6 | 16 | rs212097, rs169844, rs2376957, rs4781732, rs212072, rs8058694, rs8058696, rs12931472, rs3851722, rs2238469, rs2239322, rs8056397, rs4780599, rs9930886 | 14 |
| ABCC8 | 11 | rs739689, rs2074311, rs2067043, rs2237992, rs2355017, rs4148626, rs4148625, rs2237991, rs2299639, rs2299638, rs2237988, rs2237984, rs10832786, rs1799854, rs2283262, rs2074308, rs4148618, rs7106053, rs722341, rs2283255, rs11024300, rs1048099, rs3758947, rs985572 | 24 |
| ABCC9 | 12 | rs829060, rs2638441, rs16924332, rs1283809, rs11046202, rs704175, rs704179, rs829080, rs1283822, rs4148674, rs7966768, rs697252, rs704205, rs7301876, rs1356368, rs10743426, rs2277404, rs11046238 | 18 |
| ABCD1 | X | rs5987140 | 1 |
| ABCD2 | 12 | rs7968837, rs4294600, rs6581208, rs12227668, rs10877201 | 5 |
| ABCD3 | 1 | rs10493872, rs12750904, rs1041282, rs17410643 | 4 |
| ABCD4 | 14 | rs2301345 | 1 |
| ABCE1 | 4 | rs1378315, rs17725600 | 2 |
| ABCF1 | 6 | rs3130041, rs2269710, rs1264440, rs1264437, rs1264432 | 5 |
| ABCF2 | 7 | rs310583, rs2608291, rs6967282, rs1122979, rs4726007 | 5 |
| ABCF3 | 3 | rs11539876, rs1709624, rs7614381, rs843337 | 4 |
| ABCG1 | 21 | rs8131660, rs1117640, rs225440, rs915847, rs225374, rs225376, rs7281345, rs225385, rs17767083, rs225398, rs532345, rs482303, rs2234718, rs2839482, rs492338, rs3788007, rs450808, rs2234721, rs9975333, rs1044317 | 20 |
| ABCG2 | 4 | rs2725270, rs2231164, rs12505410, rs2622621, rs13120400, rs2725252, rs6857600, rs2622626, rs2622604 | 9 |
| ABCG4 | 11 | rs4301800, rs626776, rs674424, rs4148173 | 4 |
| ABCG5 | 2 | rs2278356, rs10205816, rs4245786, rs4289236, rs4148179 | 5 |
| ABCG8 | 2 | rs4148202, rs6720518, rs4299376, rs4077440, rs4148217, rs6756778, rs4245794, rs4952689, rs13405698, rs4953028, rs4148221, rs4148222, rs6544718, rs2954804, rs2954805, rs4953029 | 16 |
| SLC10A1 | 14 | rs17556915, rs11624523, rs4646285, rs11623504, rs10130926, rs12882299 | 6 |
| SLC10A2 | 13 | rs16961116, rs3803258, rs2301159, rs279941, rs17273914, rs1925978, rs190716, rs279913, rs157266, rs3783238, rs9586056, rs16961277, rs7987433, rs3759503 | 14 |
| SLC12A1 | 15 | rs12907018, rs11070629, rs2279366 | 3 |
| SLC12A3 | 16 | rs12932041, rs12599065, rs13306673, rs4329913, rs2289119, rs8043560, rs11076172, rs2278490, rs12445698, rs7187932, rs6499858, rs11640308, rs7204044, rs1138429, rs2099107, rs2399594, rs28168, rs9921780 | 18 |
| SLC12A4 | 16 | rs7200210 | 1 |
| SLC13A1 | 7 | rs11772595, rs10281118, rs10258700, rs11976455, rs2140516, rs3824025, rs17544071 | 7 |
| SLC15A1 | 13 | rs3783001, rs6491437, rs9584911, rs4646215, rs1331251, rs4646212, rs7998137, rs4772131, rs12853199, rs7320943 | 10 |
| SLC15A2 | 3 | rs1881995, rs1523519, rs2293616, rs2257212, rs1143670, rs1143671, rs3817599, rs4285028 | 8 |
| SLC16A1 | 1 | rs12727968, rs7169, rs1049434, rs3789592, rs7552903 | 5 |
| SLC17A6 | 11 | rs7126852, rs2665691, rs11026529, rs2290045, rs1155331, rs1900586, rs10833748, rs2593685, rs11026539 | 9 |
| SLC17A7 | 19 | rs1043558, rs3826718, rs1320301, rs11672810, rs1578944 | 5 |
| SLC17A8 | 12 | rs7311178, rs10778050, rs11110356, rs7485480, rs12826643, rs17030132, rs11568537 | 7 |
| SLC19A1 | 21 | rs2330183, rs3939250 | 2 |
| SLC22A1 | 6 | rs1867351, rs461473, rs594709, rs9457843, rs2197296, rs622342, rs9295125, rs609468, rs651164, rs4646283, rs6455682, rs4709403 | 12 |
| SLC22A2 | 6 | rs10755577, rs316002, rs17588242, rs2279463, rs3127573, rs492453, rs316025, rs316026 | 8 |
| SLC22A3 | 6 | rs512077, rs641990, rs377551, rs2457556, rs1510228, rs1567441, rs10499314, rs12194182, rs2457571, rs2457574, rs1810126 | 11 |
| SLC22A4 | 5 | rs6860806, rs272886, rs1050152 | 3 |
| SLC22A5 | 5 | rs2631370, rs2073643, rs11746555, rs274548 | 4 |
| SLC22A6 | 11 | rs10897310, rs3017670, rs4149172 | 3 |
| SLC22A7 | 6 | rs1574430, rs4149178 | 2 |
| SLC22A8 | 11 | rs955434, rs10792367, rs2276299, rs4149182, rs2187383, rs4963326, rs3809069, rs948979, rs3948869 | 9 |
| SLC25A27 | 6 | rs3757241, rs9381468 | 2 |
| SLC26A8 | 6 | rs851027, rs10456082 | 2 |
| SLC28A1 | 15 | rs2242048, rs8025045 | 2 |
| SLC28A2 | 15 | rs16941017, rs10519018, rs12913645, rs16941238 | 4 |
| SLC28A3 | 9 | rs7853758, rs4585823, rs10868137, rs10868141, rs10868142, rs13288052, rs11140507, rs10746739, rs11140519, rs10735568, rs11140525, rs12004882, rs4877845, rs4877848, rs7043257, rs3812509, rs17087144, rs11140544, rs10868152, rs12378361 | 20 |
| SLC29A1 | 6 | rs9357436, rs1057985, rs693955, rs324148, rs760370, rs6458375, rs666462, rs6905285, rs9472236 | 9 |
| SLC29A2 | 11 | rs2279861 | 1 |
| SLC29A3 | 10 | rs10999776, rs6480513, rs12252139, rs2066210, rs780659, rs780660, rs780662, rs3781316, rs1110399, rs3781317, rs883764, rs780664, rs3781326, rs703256, rs2487068, rs780680, rs780689, rs780690 | 18 |
| SLC29A4 | 7 | rs6958502, rs6965716, rs3889348 | 3 |
| SLC2A1 | 1 | rs4658, rs3820548, rs3768029, rs841858, rs1385129, rs751210, rs17387775 | 7 |
| SLC30A6 | 2 | rs177083 | 1 |
| SLC35B2 | 6 | rs1875324, rs520639 | 2 |
| SLC35E3 | 12 | rs10878864 | 1 |
| SLC39A13 | 11 | rs2293577 | 1 |
| SLC39A6 | 18 | rs1107840 | 1 |
| SLC39A7 | 6 | rs1547387 | 1 |
| SLC44A4 | 6 | rs577272, rs644774, rs494620, rs2242665, rs3130481, rs614549, rs605203 | 7 |
| SLC6A18 | 5 | rs12513872, rs10078761 | 2 |
| SLC6A2 | 16 | rs36030, rs747107, rs1532701, rs192303, rs734980, rs13333066, rs36024, rs187714, rs36023, rs36020, rs16955591, rs3785152, rs40147, rs36016, rs10521329, rs5569, rs2242447, rs10521330, rs16955708 | 19 |
| SLC6A3 | 5 | rs12516758, rs27072, rs40184, rs11133767, rs6347, rs27048, rs37022, rs2042449, rs2975292, rs464049, rs460000, rs4975646, rs403636 | 13 |
| SLC6A4 | 17 | rs11080121, rs2066713 | 2 |
| SLC6A7 | 5 | rs2240784, rs11749558, rs4705430, rs10076748, rs13153325, rs4705431, rs9800032 | 7 |
| SLC7A5 | 16 | rs12929670, rs1060253, rs3889228, rs16943320, rs9938601, rs4843717, rs876985, rs6540092, rs4240803, rs4465613 | 10 |
| SLC7A7 | 14 | rs1805061, rs1805059, rs2281677 | 3 |
| SLC9A3R2 | 16 | rs2531210, rs3211995 | 2 |
| SLCO1A2 | 12 | rs2199680, rs11045913, rs10841781, rs10505872, rs4115170, rs4078, rs10743411, rs2857468, rs4762818, rs4762699, rs10841801, rs11046012, rs7316461 | 13 |
| SLCO1B1 | 12 | rs4149022, rs11045787, rs7138177, rs4149032, rs4149033, rs2291073, rs2291075, rs6487213, rs999278, rs2417963, rs11045834, rs12317268, rs10841763, rs10841767, rs12371604 | 15 |
| SLCO1B3 | 12 | rs1356148, rs10841661, rs11045586, rs10770757, rs2117032 | 5 |
| SLCO2B1 | 11 | rs4944074, rs10501421, rs1612859, rs1077858, rs10793116 | 5 |

**Supplementary Table ST2. ABCG2 haplotype distribution.** ABCG2 haplotype at loci rs12505410, rs13120400 and rs2725252 were determined independently in the three populations analyzed. Haplotyping was performed using the EM algorithm implemented in the Haplostat R library.^31^

|  |  | **Population** | | |
| --- | --- | --- | --- | --- |
| **#** | **Haplotype** | **SLEC** | **CEU** | **SVC** |
| Hap. 1 | G-C-G | 22.28 | 25 | 27.65 |
| Hap. 2 | G-C-T | 0.47 | 0.66 | 0 |
| Hap. 3 | G-T-G | 5.06 | 3.48 | 6.29 |
| Hap. 4 | T-T-G | 14.59 | 10.34 | 11.91 |
| Hap. 5 | T-T-T | 56.03 | 54.8 | 51.47 |
| Hap. 6 | G-T-T | 1.58 | 5.73 | 2.49 |
| Hap. 7 | T-C-G | 0 | 0 | 0.2 |

**Supplementary Figure SF1.**

**Supplementary Figure SF1. Cumulative incidence of MMR at 18 month according to ABCG2 genotypes from the three validated SNPs** (rs12505410, rs13120400 and rs2725252) **according to imatinib dose (400 mg/d or 600 mg/d) in the SVC.**
